# Health-promoting work schedules among nurses and nurse assistants in France: results from nationwide AMADEUS survey

**DOI:** 10.1186/s12912-023-01403-9

**Published:** 2023-08-03

**Authors:** Guillaume Fond, Guillaume Lucas, Laurent Boyer

**Affiliations:** 1 CEReSS-Health Service Research and Quality of Life Center, Assistance Publique des Hôpitaux de Marseille, Aix-Marseille University, 27, boulevard Jean -Moulin, Marseille, 13005 France; 2 Fondation FondaMental, Créteil, France

**Keywords:** Public health, Mental health, Nursing, burnout, Work schedule

## Abstract

**Background:**

The study aimed to investigate the relationship between different work schedules and self-reported working conditions and health risk behaviours among nurses and nurse assistants (NNA) in France. It hypothesized that work schedules, particularly long shifts, could impact work-life balance, workload, stress levels, burnout, and smoking habits. NNA had the option to work either with a 7-hour schedule, 5 days per week, or with long work schedules consisting of ten to twelve-hour shifts, three days per week. These schedules could potentially influence various aspects of their professional lives.

**Methods:**

The survey followed the guidelines of the Strengthening the Reporting of Observational Studies in Epidemiology (STROBE) Statement and was administered to NNA working in public and private national healthcare facilities in France. The researchers used the Job Content Questionnaire to assess the work environment and the French version of the 22-item Maslach Burnout Inventory (MBI) scale to measure burnout.

**Results:**

A total of 3,133 NNA participated in the study, including 2,369 nurses (75.6%) and 764 nurse assistants (24.4%). Among them, 1,811 individuals (57.8%) followed a 7-hour work schedule, while 1,322 individuals (42.2%) had a long work schedule. Multivariate analyses revealed that NNA working with long schedules reported higher psychological demands, more frequent burnout, a higher number of daily smoked cigarettes, and greater coffee consumption. These findings were independent of other factors such as sector of employment, type of healthcare facility, job status, work schedules, night shifts, department specialty, age, and family responsibilities.

**Conclusions:**

While some NNA may choose long schedules to have more days off, those working with these schedules experience greater work-related burdens and engage in worse health risk behaviours as a coping mechanism. It emphasizes the importance of considering health-promoting work schedules to address the high psychological demands and burnout experienced by NNA with long schedules. Implementing changes in work schedules could potentially improve the overall well-being and job satisfaction of these healthcare professionals.

**Supplementary Information:**

The online version contains supplementary material available at 10.1186/s12912-023-01403-9.

## Introduction

Health-promoting work schedules have gained attention as a means to improve the physical and mental well-being [1] and enhance the retention of nurses and nurse assistants [2]. Numerous studies conducted during the Covid-19 crisis have highlighted the prevalence of sleep disorders among NNA, further emphasizing the importance of health-promoting schedules [3]. A recent systematic review has indicated the need for research on interventions aimed at managing fatigue in nurses [4].

Excessive working hours can lead to stress and sleep disorders, increasing the risk of chronic fatigue and illnesses, including infections [5]. It is now established that working more than 40 h per week and engaging in 12-hour shifts is associated with adverse patient outcomes [6]. In the intensive care units of two hospitals, over half of the nurses working 12-hour shifts reported experiencing low to moderate levels of chronic fatigue [7]. European registered nurses working shifts of 12 h or longer and those working overtime have reported lower quality and safety levels and a higher incidence of unfinished tasks [8]. As a result, some researchers have suggested reconsidering the use of 12-hour work schedules [9].

However, an older study published in 2006, comparing 8-hour and 12-hour schedules in thirteen New York City hospitals, found that nurses working 12-hour shifts reported higher job satisfaction without a decrease in the quality of care provided [10]. Long work schedules, such as 12-hour shifts, may also lead to reduced commuting time, potentially resulting in lower fatigue levels. A recent study conducted with a small sample of 48 nurses suggested that individual preferences for 12-hour shifts are influenced by factors such as personal health, family situation, tolerance for workload, sleep issues, personality, and other variables [11]. The authors concluded that, to attract and retain nurses, individuals should be given the freedom to choose 12-hour shifts.

In summary, it is evident that 12-hour work schedules are associated with various negative outcomes. However, it is crucial to note that no specific studies have been conducted in France to explore this relationship. Moreover, previous studies may not have adequately considered certain confounding factors, such as the presence of a partner and children at home, which could potentially impact the risk of emotional exhaustion and sleep deprivation, among other factors. A study conducted in South Africa with 71 nurses identified household factors as important contributors to emotional exhaustion [12]. In Korea, a work-family-school role conflicts model explaining burnout was validated with 286 nurses [13]. In France, the healthcare system is divided into medical and medico-social sectors, and into public and private sectors, each potentially introducing confounding factors regarding the associations between work schedules and adverse outcomes.

The objective of this study was to identify work schedules associated with self-reported improved working conditions and health risk behaviours among NNA.

## Population and methods

### Study population and study design

The method followed the Strengthening the Reporting of Observational Studies in Epidemiology (STROBE) Statement guidelines[14].

#### Design

The AMADEUS (« *AMéliorer l’ADaptation à l’Emploi pour limiter la soUffrance des Soignants* »/ « *improve employment adaptation to limit caregiver stress* ») study is a cross-sectional survey carried out in French public and private healthcare facilities at a national level in France between May 2, 2021 and June 30, 2021. This survey was supported by professional healthcare worker associations and the directions of the healthcare settings in which the survey was disseminated. The detailed protocol has been published [15]. Recruitment and sampling method, inclusion and exclusion criteria are presented in Supplementary Annex 1.

### Collected data

#### Sample characteristics

The following job characteristics were recorded as binary variables: Nurse (vs. Nurse assistant), Public sector (vs. private), Hospital (vs. medico-social facility), Full-time job, Constant schedules, Planned schedules (defined by the actual working schedule differing from the roster over the past two weeks), Night shift job. The reported departments included Surgery, Medical specialty, Psychiatry, and Critical care.

The following individual characteristics were recorded: Sex (male/female), Age (years), Partnership status, and Having children at home (all binary except age, which is continuous). It is worth noting that having children at home has been identified as a significant predictor of work-related stress and burnout among midwives [16].

#### Work environment

The work environment was assessed using the Job Content Questionnaire (JCQ) [17]. The JCQ examined three axes, measured by 26 items, which were treated as continuous variables. Axis 1 represented high psychological demand, encompassing speed and quantity (3 items), complexity and intensity (3 items), and fragmentation and unpredictability (3 items). Axis 2 represented low decisional latitude, including decision-making latitude (3 items), use of skills (3 items), and skill development (3 items). Axis 3 represented low social support, encompassing professional support by superiors (2 items), professional support by colleagues (2 items), emotional support by superiors (2 items), and emotional support by colleagues (2 items). All factor dimensions exhibited satisfactory Cronbach’s alpha coefficients ≥ 0.7 [18].

In a study conducted with Swedish workers using the Swedish version of the JCQ, Cronbach’s alpha scores of 0.71 were reported for psychological demand, 0.82 for decisional latitude, and 0.79 for social support [19]. Similarly, a study involving Japanese workers using a translated version of the JCQ reported Cronbach’s alpha scores of 0.76 for psychological demand, 0.81 for decisional latitude, and 0.85 for social support [20]. Convergent validity tests confirmed the expected associations with key variables such as age, work status, sector of activity, occupation, job satisfaction, perception of job stress, and intent to change jobs [18].

#### Burnout

The dimensions of burnout syndrome were assessed as binary variables using the French version of the 22-item Maslach Burnout Inventory (MBI) scale, following the recommendations of the validation study [21]. The 22-item scale has demonstrated satisfactory psychometric properties [22]. The stability coefficients were reported as 0.82 at 2–4 weeks and 0.80 at one year, indicating good reliability [22].

#### Health risky behaviours

Number of daily smoked cigarettes and coffee consumption (cups/day) were reported as continuous variables.

### Statistical analysis

All variables were presented using frequency distributions for categorical variables and mean (standard deviation) for continuous variables. Comparisons between nurses and nurse assistants working in 7-hour schedules versus long schedules were conducted using the chi-square test for categorical variables. Parametric continuous variables were analysed using Student’s t-tests, while non-parametric continuous variables were analysed using Mann-Whitney tests, depending on the distribution of the data. Associations with a p-value < 0.05 were considered statistically significant and included as adjustment factors in the multivariate models.

In the multivariate analyses, logistic regression models were used for binary variables (burnout), and linear regression was employed for continuous variables (psychological demand/Axis 1, number of daily smoked cigarettes, and coffee consumption). Each model was adjusted for variables that exhibited significant differences between the 7-hour work schedule and the 12-hour work schedule in the univariate analyses (p-value < 0.05). Data analysis was performed using SPSS 20.0 software.

## Results

The sample characteristics are presented in Table 1. Overall, 3133 nurses and nurse assistants were recruited: 2369(75.6%) nurses and 764(24.4%) nurse assistants, of those 1811(57.8%) (1366 nurses and 445 nurse assistants) had a 7-hour schedule and 1322(42.2%) (1003 nurses and 319 nurse assistants) had a long schedule. The following variables were significantly different (p < 0.05) between nurses and nurse assistants working with 7-hour schedule vs. long schedule: working in public sector (vs. private); working in hospital (vs. medico-social facility), having a full-time job, having constant schedules, planned schedules, a night shift job, working in medical specialty department, psychiatry department, critical care department and individual characteristics (age, being partnered, having children at home).

**Table 1:** Sample characteristics

	Whole sample (N = 3133)			7-hour schedule (N = 1811)			Long work schedule (N = 1322)						
	N or mean	% or SD		N or mean	% or SD		N or mean		% or SD		P value		
Job characteristics													
Nurse(N,%)	2369	0.756		1366	0.754		1003		0.759		0.776		
Nurse assistant(N.%)	764	0.244		445	0.246		319		0.241				
Public sector (vs. private) (N,%)	2754	0.879		**1573**	**0.869**		**1181**		**0.893**		**0.036**		
Hospital (vs. medico-social facility) (N,%)	2948	0.941		**1681**	**0.928**		**1267**		**0.958**		**< 0.001**		
Full-time job(N,%)	2589	0.826		**1472**	**0.813**		**1117**		**0.845**		**0.019**		
Constant schedules(N,%)	1504	0.480		**680**	**0.375**		**824**		**0.623**		**< 0.001**		
Planned schedules(N,%)	2874	0.917		**1636**	**0.903**		**1238**		**0.936**		**0.001**		
Night shift job(N,%)	494	0.158		**15**	**0.080**		**479**		**0.362**		**< 0.001**		
Departments													
Surgery(N,%)	511	0.163		290	0.160		221		0.167		0.598		
Medical specialty(N,%)	1714	0.547		**916**	**0.506**		**798**		**0.604**		**< 0.001**		
Psychiatry(N,%)	513	0.164		**411**	**0.227**		**102**		**0.077**		**< 0.001**		
Critical care(N,%)	406	0.130		**109**	**0.060**		**297**		**0.225**		**< 0.001**		
Individual characteristics													
Sex (man) (N,%)	453	0.145		261	0.144		192		0.145		0.930		
Age (years) (mean(SD)	**40.88**	**10.15**		**41.52**	**10.02**		**39.24**		**10.28**		**< 0.001**		
Partnered(N,%)	2292	0.732		**1350**	**0.745**		**942**		**0.713**		**0.040**		
Children at home(N,%)	1952	0.623		**1167**	**0.644**		**785**		**0.594**		**0.004**		

Significant associations are in bold (p < 0.05). SD standard deviation

The Cronbach alpha coefficients of the Job content questionnaire were respectively 0.791 for psychological demand (Axis 1), 0.583 for decisional latitude (Axis 2) and 0.725 for social support (Axis 3). The univariate analyses of the associations between job outcomes and work schedules are presented in Table 2 and multivariate analyses in Table 3. One model was carried out for each work outcome variables that was significantly different between groups in univariate analyses (psychological demand /Axis 1, burnout, number of daily smoked cigarettes, coffee consumption). After adjustment, nurses and nurse assistants working with long schedules reported significantly higher psychological demand (Axis 1) (Beta (B) = 1.108 95% confidence interval (CI)[0.693 ;1.522], p < 0.001), burnout (adjusted odds ratio (aOR) = 1.221, 95%CI[1.024;1.456], p = 0.026), number of daily smoked cigarettes (B = 0.760, 95%CI[0.291;1.229], p = 0.001) and coffee consumption (B = 0.255, 95%CI[0.030;0.479], p = 0.026).

**Table 2:** Associations between healthcare workers working with long work schedules vs. 7-hour schedules and work environment (univariate analyses)

	7-hour schedule (N = 1811)		Long work schedule (N = 1322)			
	N or mean	% or SD	N or mean	% or SD		p
Psychological demand (Axis 1, mean/SD)	**25.76**	**5.02**	**26.40**	**4.91**		**< 0.001**
Decisional latitude (Axis 2, mean/SD)	70.25	10.89	69.97	9.95		0.256
Social support (Axis 3, mean/SD)	23.22	4.52	22.98	4.45		0.106
Burnout(N,%)	**976**	**0.539**	**760**	**0.575**		**0.046**
Number of daily smoked cigarettes	**2.42**	**5.21**	**2.99**	**5.84**		**0.005**
Coffee consumption	**2.53**	**2.50**	**2.66**	**2.81**		**0.077**

Significant associations are in bold (p < 0.05). SD standard deviation

**Table 3:** Associations between healthcare workers working with long work schedules vs. 7-hour schedules and work environment, burnout and health risky behaviours (dependent variables): multivariate analyses*

	aOR or B	95%CI	P
Axis 1 psychological demand (B)	**1.108**	**0.693 ;1.522**	**< 0.001**
Burnout (aOR)	**1.221**	**1.024;1.456**	**0.026**
Number of daily smoked cigarettes (B)	**0.760**	**0.291;1.229**	**0.001**
Coffee consumption (B)	**0.255**	**0.030;0.479**	**0.026**

* One model was carried out for each variable. All models were adjusted for the following independent variables: profession, department and individual characteristics that were significantly different between groups in univariate analyses (i.e. Public sector (vs. private), Hospital (vs. medico-social facility), Full-time job, Constant schedules, Planned schedules, Night shift job, Medical specialty, Psychiatry, Critical care, Age, Partnered, Children at home)

aOR adjusted odds ratio. 95%CI : 95% confidence interval; B: non-standardized Beta, SE: standard error

All models were adjusted for job, department and individual characteristics that were significantly different between groups in univariate analyses (i.e.: Public sector (vs. private), Hospital (vs. medico-social facility), Full-time job, Constant schedules, Planned schedules, Night shift job, Medical specialty, Psychiatry, Critical care, Age, Partnered, Children at home).

## Discussion

Consistently with most of the previous studies, French nurses and nurse assistants working long schedules reported higher psychological demand, higher rates of burnout, and worse health risky behaviours compared to those working a 7-hour schedule.

Nursing is a profession known for its high demands, intense workload, and challenging work environment. Nurses and nurse assistants often work long and irregular schedules, which can negatively impact their mental and physical health. Numerous studies have shown that long work hours and shift work can increase the risk of various health problems, including burnout, psychological distress, and sleep disorders.

For example, in Nigeria, seven focus groups consisting of 66 nurses reported that long work hours and burnout were factors leading to job cessation [23]. Similarly, a survey of 2428 nurses from 32 hospitals in Malaysia found that one in four nurses reported burnout, and shift working, double-shifts, and night shifts were associated with burnout [24]. A study conducted in Taiwanese nurses yielded similar results, indicating that long working hours and night shifts were associated with increased psychological distress [25]. In the United States, a study involving 2488 nurses reported low-to-moderate intershift recovery associated with increased burnout, and the length of shifts was associated with well-being indices [26]. Another US survey with 318 nurses found that hours worked was one of the factors associated with emotional exhaustion [27]. In China, nurses working long hours experienced more severe secondary traumatic stress [28]. A recent review of literature on emergency department nursing burnout also reported an association between work schedules and increased burnout [28]. In a survey of 2744 healthcare workers in Singapore, working long hours was associated with higher odds of burnout and stress, while teamwork and feeling appreciated at work were associated with lower odds of stress, anxiety, and job burnout [29]. These results were confirmed in a longitudinal study of 247 nurses in the Netherlands, where work schedule predicted emotional exhaustion at one year [30]. A Cochrane systematic review found low evidence suggesting that changing work schedules may lead to a reduction of stress [31]. These observational data provide important findings to guide health policies and prevention.

In our study, we found that nurse assistants working long schedules reported higher smoking and coffee consumption. A meta-analysis including 243 published records [32] revealed that long working hours were associated with high psychological stress, work stress, and increased smoking (coffee consumption was not explored). Smoking and coffee consumption may be considered coping strategies for nurse assistants to deal with burnout and high psychological demand. It is possible that nurse assistants working long schedules have more breaks during their shift, which may lead them to consume more coffee and cigarettes (for smokers) to increase alertness, concentration, and reduce stress. Prevention programs could provide information about the long-term risks of such behaviours, particularly regarding mental health, which is less well-known compared to the risks for cardiovascular diseases and cancer [33]. Promoting breaks that provide full opportunities for rest, such as napping at work and mindfulness, is a promising strategy to help nurse assistants manage work-related stress without relying on coffee and tobacco consumption [34]. Based on our results, prioritizing interventions to improve these health risky behaviours should focus on nurse assistants working long schedules (12 h) [35, 36].

Limitations and perspectives: There are two important missing pieces of data in the AMADEUS study: whether nurse assistants have the ability to choose their schedule and their chronotype pattern [37]. Our results have shown that long work schedules are more prevalent in medical specialties and critical care, while 7-hour schedules are more common in psychiatry. Nurse assistants may have to choose between the schedule and the department in which they work, especially if they have the option, which is not always the case, particularly for neoprofessionals. Nurse assistants with an evening chronotype pattern may report better job satisfaction with evening shifts [37]. Qualitative research could also explore the expectations of nurse assistants and managers. Job satisfaction was not reported in the present study. The Cronbach alpha coefficient for decisional latitude was < 0.7, which limits its interpretation as a measure of the construct in this study. As salaries for nurses in France are fixed, they were not included in the survey. Quick return (defined as less than 11 h of rest time between two shifts) [1] was not reported in the present study since 11 h is the minimum rest time mandated for all nurse assistants according to French law and the EU’s Working Time Directive (2003/88/EC) [38]. The limitations of this study are similar to those affecting other online surveys. Disseminating the study to nurses and nurse assistants was less effective due to the lack of access to professional mailing lists in some facilities. However, the survey was disseminated through social networks. To mitigate selection bias, the study’s title did not explicitly mention work schedules or burnout but instead focused on work adaptation. In France, we have no direct access to professional listings and/or mailing lists, which limits the implementation of surveys. Therefore, there is a need for nationally representative cohorts to monitor the work environment and health status of nurse assistants.

## Conclusion

Long work schedules are associated with worse outcomes including higher psychological demand, higher burnout and higher health risky behaviours to cope with fatigue and stress.

### Electronic supplementary material

Below is the link to the electronic supplementary material.

Supplementary Material 1

## Data Availability

The datasets used and/or analysed during the current study available from the corresponding author on reasonable request.

## Data Availability

Data are available on reasonable request to the authors.
