# Supplementary material for: Health-promoting work schedules among nurses and nurse assistants in France: results from nationwide AMADEUS survey
Source: BMC Nurs. 2023 Aug 3;22:255. doi: 10.1186/s12912-023-01403-9 (PMC10399037; doi:10.1186/s12912-023-01403-9)
Supplement: Supplementary file 1 — Supplementary Material 1 [file 12912_2023_1403_MOESM1_ESM.docx]

Supplementary Annex 1. Recruitment process.

*Recruitment and sampling method.* The participants were contacted through public and private facilities and professional associations by professional mailings and through social networks. All professional associations were contacted by email, and all regional health agencies, Territory hospital groups (*Groupements hospitaliers de territoire* /GHT including regional networks of academic and non-academic public hospitals) were contacted by phone to increase participation rates.

The following institutions/facilities actively participated in the dissemination of the study:

-(Regional health agencies) Provence Alpes Côte d'Azur, Brittany, Ile de France;

-(Territory hospital groups including academic and non-academic public hospitals) Alps, Dauphiné, Rhône center, South Drôme, Ardèche, Western Brittany, South Brittany, Upper Brittany, South Corsica, South Val d'Oise, North Hauts-de-Seine, Ile de France, South Vaucluse, Var, Alpes-de-Haute-Provence, Bouches-du-Rhône, Alpes Maritimes, Southern Alps;

-(Academic hospitals): XX, Assistance publique Hôpitaux de Paris, Hospices civils de Lyon, CHU d 'Amiens, CHU d 'Angers, CHU de Besancon, CHU de Brest, CHRU de Caen, CHU de Clermont-Ferrand, CHU de Dijon, CHU de Grenoble, CHU of Lille, CHU of Limoges, CHU of Martinique, CHU of Montpellier, CHRU of Nancy, CHU of Nantes, CHU of Pointe-à-Pitre/Les Abymes, CHU of Reims, CHU of Rennes, CHU of La Reunion, CHU of Rouen, CHU of Saint-Étienne, CHU of Toulouse, CHRU of Tours;

-(Private hospitals) Institut de cancérologie de l'Ouest, Institut Bergonié, Centre François Baclesse, Centre Jean Perrin, Centre George-François Leclerc; Centre Oscar Lambret; Centre Léon Bérard; Institut Paoli-Calmettes; Centre Antoine Lacassagne, Centre d’Oncologie et de Radio Thérapie 37 (CORT37)

-(Associations) National Association of Graduate Nurses and Students, French association of care managers, Committee of agreement of the nursing training and executives, Fédération hospitalière de France, Fédération nationale des associations d'aides-soignants, Syndicat national des infirmiers anesthésistes, Syndicat national des infirmiers de bloc opératoire.

Finally, the survey was disseminated through social networks at two timepoint (at the beginning and one month later) to ensure the maximum representativeness of the sample.

*Inclusion criteria.* The participants were graduated HCWs currently working in a French public or private health facility.

*Exclusion criteria.* As the purpose was to evaluate work environment, HCWs working in their private office were not included.
